# Psychophysiological reactivity, coping behaviour and intrusive memories upon multisensory Virtual Reality and Script-Driven Imagery analogue trauma: A randomised controlled crossover study

**DOI:** 10.1016/j.janxdis.2018.08.005

**Published:** 2018-10

**Authors:** Tina Schweizer, Fritz Renner, Dali Sun, Birgit Kleim, Emily A. Holmes, Brunna Tuschen-Caffier

**Affiliations:** a Department of Psychology, Clinical Psychology and Psychotherapy, University of Freiburg, 79085 Freiburg, Germany; b MRC Cognition and Brain Sciences Unit, University of Cambridge, CB2 7EF Cambridge, United Kingdom; c Department of Computer Science, University of Freiburg, 79110 Freiburg, Germany; d Department of Experimental Psychopathology and Psychotherapy, University of Zurich, 8050 Zurich, Switzerland; e Department of Psychiatry, Psychotherapy and Psychosomatics, Psychiatric Hospital, University of Zurich, 8032 Zurich, Switzerland; f Division of Psychology, Department of Clinical Neuroscience, Karolinska Institutet, SE-171 65 Solna, Sweden

**Keywords:** Risk factors, Psychopathology, Post-traumatic stress, Stress and coping measures, Guided mental imagery, Virtual Reality

## Abstract

**Background:**

Peri- and post-traumatic factors predict the differential development of stress-associated mental disorders. Prospective designs assessing these risk factors in real-time under controlled experimental conditions can overcome limitations of retrospective designs. Therefore, we aimed to investigate multi-sensory, experimental analogues of a traumatic experience delivered in Virtual Reality (VR) or Script-Driven Imagery (SDI).

**Methods:**

In a randomised controlled crossover design, differences in the induced analogue trauma symptoms between multi-sensory analogue trauma by either VR or SDI versus a neutral condition were assessed in 127 non-clinical participants.

**Results:**

Analogue symptoms (psychophysiological responses, coping behaviour and intrusive memories of the experimental trauma) increased following analogue trauma in both VR and SDI, with more analogue symptoms for VR. Psychophysiological arousal was in general higher in VR.

**Limitations:**

The analogue trauma situation of a car park fire that was used may be infrequent in real life.

**Conclusions:**

Multisensory (vision, olfaction, hearing) analogue trauma in VR and SDI offers a useful tool for the induction and real-time assessment of peri- and post-traumatic risk factors for analogue stress-associated psychopathology. VR was more effective in inducing analogue symptoms than SDI, even though the latter might be more personalised. New experimental models for studying trauma exposure and responses may contribute to a better understanding of risk factors and help to identify and protect individuals at risk.

## Highlights

• Real-time assessment of peri- and post-traumatic risk factors for stress disorders.
• Experimental stress induction by a VR and SDI multisensory analogue trauma paradigm.
• VR and SDI induced trauma-like symptoms in a randomised controlled crossover design.
• VR induced more trauma-like symptoms than SDI.
• Both paradigms offer real-time modelling of stress-associated disorders.

## 1 Introduction

After experiencing a traumatic event, not all individuals develop stress-associated disorders such as post-traumatic stress disorder (PTSD) or depression (Bonanno, Westphal, & Mancini, 2011). Pre-existing psychophysiological markers such as higher resting heart rate (HR) (Bryant et al., 2000, Bryant et al., 2003), higher skin conductance (SC) and slower SC habituation to startling sounds (Orr et al., 2012; Pole et al., 2009) are associated with an increased risk to develop PTSD. However, key factors that modulate the differential course of PTSD development include those that operate during and directly after the traumatic event, such as peri-traumatic emotional responses (Badour & Feldner, 2013; Ozer, Best, Lipsey, & Weiss, 2003), coping behaviour (Gamberini, Cottone, Spagnolli, Varotto, & Mantovani, 2003; Saxon et al., 2017) and early intrusive memories (Michael, Ehlers, Halligan, & Clark, 2005; Solberg, Birkeland, Blix, Hansen, & Heir, 2016) and appraisals (Speckens, Ehlers, Hackmann, & Clark, 2006). Previous studies investigating these risk factors have mainly used retrospective assessments that are prone to memory biases (Cortese, Leslie, & Uhde, 2015; Halligan, Clark, & Ehlers, 2002). To assess risk factors in real-time, prospective designs using analogue trauma for stress induction under controlled experimental conditions are needed.

Among the most established paradigms to induce stress in a laboratory setting are viewing aversive static pictures (e.g. International Affective Picture System (IAPS); (Lang, Bradley, & Cuthbert, 2005; Oulton, Takarangi, & Strange, 2016) and viewing traumatic film footage (Holmes & Bourne, 2008; James et al., 2016), both eliciting comparable stress levels (Uhrig et al., 2016). However, these paradigms may rely considerably on individuals’ capacity for perspective taking and empathising with other people (Davis, Hull, Young, & Warren, 1987; van der Heiden, Scherpiet, Konicar, Birbaumer, & Veit, 2013). Paradigms providing the opportunity to experience events from a *first-person perspective* may overcome this limitation (Bach, Fenton-Adams, & Tipper, 2014; Kaufman & Libby, 2012; McIsaac & Eich, 2004). For example, they may enable the generation of new personal memories with high self-relevance (Brown, Keenan, & Potts, 1986; Hyman & Pentland, 1996; Schöne, Wessels, & Gruber, 2017) and person-environment interactions (Lang, 1979; McMillan, 2002; Rovira, Swapp, Spanlang, & Slater, 2009), notably including coping behaviour (Arble, Lumley, Pole, Blessman, & Arnetz, 2017; Tammy Lin, 2017).

Virtual Reality (VR) is a promising experimental stress induction method allowing for first-person perspective experiences (Becker-Asano et al., 2011; Dibbets & Schulte-Ostermann, 2015; Schweizer et al., 2017). In VR, movements with real-time visual-auditory orientation and 3-D views are enabled by a joystick and a head-tracked head-mounted display. VR is associated with high levels of presence (a feeling of “really being” within VR) (Rovira et al., 2009) and immersion (Riva et al., 2007), both facilitated by multi-sensory simulations (Bordnick, Graap, Copp, Brooks, & Ferrer, 2005; Munyan et al., 2016) and person-environment interactions (Rovira et al., 2009). VR enables experimental control and the manipulation of distinct variables in complex situations (Kleim & Westphal, 2011; Rovira et al., 2009). A standardised VR application allows for stress responses to be recorded in real time (Riva et al., 2007) and comparisons between individuals while coping with stress (Kinateder et al., 2014; Rovira et al., 2009). The psychophysiological stress response qualitatively seems to share similarities to real traumatic situations but less intense, thus providing ecological validity (Kinateder et al., 2014; Rovira et al., 2009). VR has been shown to induce higher emotional stress levels than viewing of aversive pictures (Courtney, Dawson, Schell, Iyer, & Parsons, 2010) and elicits similar stress responses and intrusive memories as trauma films (Becker-Asano et al., 2011; Cuperus, Klaassen, Hagenaars, & Engelhard, 2017; Dibbets & Schulte-Ostermann, 2015).

Another standardised stress induction allowing for first-person perspective experiences (McTeague et al., 2010) is Script-Driven Imagery (SDI), where participants vividly imagine a stressful event with themselves being actively involved as described by a narrator using a standardised script. Mental imagery is defined as a perception-like experience without sensory input, and is strongly related to emotional processes and evokes more intense emotional responses than does verbal processing (Holmes & Mathews, 2010). Since imagined interaction with a stimulus can elicit similar emotional reactions as a real interaction with the same stimulus (Lang, 1979), it may function ‘as an “as-if real” template’ for practising and modifying emotional and behavioural reactions to the same stimulus in real life (Ji, Heyes, MacLeod, & Holmes, 2016). Thus, the flexible adaption to a given individual’s unique personal experience is an important strength of the SDI methodology, as one can argue that SDI can be more personalised for a given event, than would be a more standardised stimuli such as VR. Mental imagery is further strongly linked to autobiographical memory processes in general (Hassabis, Kumaran, Vann, & Maguire, 2007) and mental imagery of autobiographical traumatic events can reactivate trauma-related stress responses (McTeague et al., 2010). Similarly, imagery of standardised stressful scripts, for example of a traumatic situation, can also elicit stress responses and intrusive mental images in the absence of having experienced or witnessed such an event (Hassabis et al., 2007; Krans, Näring, Holmes, & Becker, 2010; McTeague et al., 2010).

Here we developed two standardised, multisensory analogue traumatic events to create a stress induction - one in VR and one in SDI - to investigate peri- and post-traumatic risk factors potentially modulating stress processing and disorder development, in a controlled laboratory setting. Multisensory components were added to both paradigms with the intention to make them stronger. Previous studies by our group have found that the VR paradigm can induce emotional stress comparable to viewing a 2-D trauma film and intrusive memories (Becker-Asano et al., 2011; Schweizer et al., 2017). The SDI paradigm has also been shown to elicit psychophysiological stress (unpublished results). Coping behaviour, defined as the effort to manage situations that are appraised as stressful (Lazarus, 1993), can be assessed in both paradigms. Coping behaviour is negatively associated with post-traumatic stress and trauma symptoms as well as positively with psychological well-being (Butler et al., 2005; Canter, 1996). Active (vs passive) coping, seems to be the most adaptive behaviour in situations with possible controllability (Olff, Langeland, & Gersons, 2005; Thompson, Fiorillo, Rothbaum, Ressler, & Michopoulos, 2018). This combination of real-time assessment including coping behaviour is a novel innovation in analogue trauma designs. In the current study, trait mental imagery (Johnsen & Lutgendorf, 2001) and experienced realness in both paradigms (Mathews, Ridgeway, & Holmes, 2013; Pearson, Naselaris, Holmes, & Kosslyn, 2015; Riva et al., 2007; Rovira et al., 2009), computer game experience in VR (Enochsson et al., 2004) and imagery perspective in SDI (Holmes, Coughtrey, & Connor, 2008) were also assessed.

In the current study, we used a randomised experimental crossover design to investigate and compare the stress induction effect compared to a neutral control of both using a VR and SDI as an experimental analogue of a trauma event. Both VR and SDI were multi-sensory (visual, olfactory, auditory). Outcome measures included psychophysiological emotional response (reactivity of anxiety, arousal, stress, helplessness, skin conductance, heart rate), coping behaviour (expert ratings of adaptive/maladaptive coping behaviours) and intrusive memories of the analogue traumatic event (frequency, related worry and mental occupation) in real-time. We further explored intrusive memories, hyperarousal and avoidance one week after the analogue trauma.

We hypothesised that both paradigms, VR and SDI, would induce higher psychophysiological responses in response to an analogue trauma compared to the neutral condition. In addition, we expected higher psychophysiological responses in the analogue trauma for VR than SDI. Furthermore, we expected that VR and SDI analogue trauma can both impact coping behaviours and intrusive memories, with more frequent coping behaviours and intrusive memories following VR than SDI.

## 2 Methods

### 2.1 Participants

**Fig. 1:**
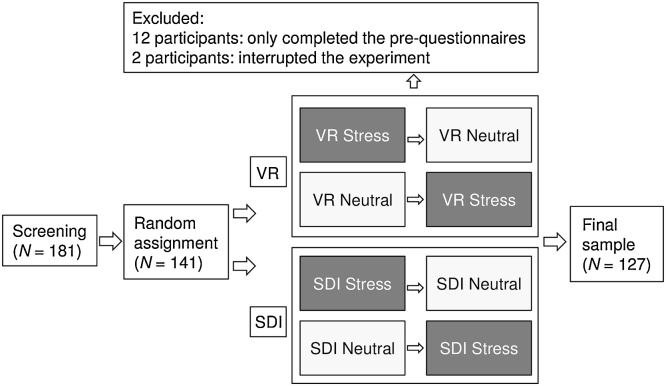
**Study flowchart.** After screening 181 participants, 141 individuals were randomly assigned to the Virtual Reality (VR) or Script-Driven Imagery (SDI) paradigms, in the order stress (analogue trauma) followed by a neutral condition or vice versa (counterbalanced). After excluding 14 participants, the final sample consisted of 127 individuals.

Participants were 141 volunteers recruited via advertisements. Exclusion criteria were a reported previous or current diagnosis of a psychological disorder, previous exposure to a traumatic event, the use of emotion-affecting medications (e.g. pain reliever). An a priori sample size calculation suggested 62 participants per group to detect a medium between-group effect size in a MANOVA (1-*β* = 0.80, *α* = 0.05). After screening 181 individuals, 141 participants were included. Participants were randomly assigned to VR or SDI, and either received a neutral or the analogue trauma condition first (counterbalanced). Fourteen participants were further excluded from analyses, as they only completed baseline questionnaires or terminated the experiment (Fig. 1).

The final sample consisted of 127 individuals (*n* = 66 VR; *n* = 61 SDI), mean age was 23 years (*M* = 22.89; *SD* = 5.92). Participants did not receive financial compensation. The study was approved by the Ethics Commission of the University of Freiburg (reference number: 221/11). All participants provided their written and informed consent.

### 2.2 Procedure

We used a mixed design with delivery method (VR, SDI) as randomly assigned between-subject factor and experimental condition (neutral condition, analogue trauma condition) as within-subjects factor in counterbalanced order by experimental condition (Fig. 1).

Participants first received information about the study, provided written informed consent and completed baseline questionnaires. Baseline levels of emotional responses (resting state) were obtained during (physiological measures: SC, HR) and after (self-report: anxiety, arousal, stress, helplessness) watching a non-arousing five-minute film clip of landscapes. Afterwards, all participants completed either VR or SDI training (according to condition allocation) and were then exposed to both the neutral and analogue trauma conditions by VR or SDI (in counterbalanced order). We used additional baselines of 5 min in between all phases to avoid carry-over effects. The VR and SDI scripts were presented with the same content until the emergency situation occurred. No instructions were given about how to behave during the emergency situation to allow a realistic simulation. During the analogue trauma, the experimenter sprayed a smell of smoke (Wilhelm Perfumes, Zürich, Switzerland) to enhance immersion and realness in the emergency scenario (Bordnick et al., 2005; Munyan et al., 2016). Following each phase, anxiety, arousal, stress and helplessness was assessed. Physiological emotional arousal was continuously recorded throughout the experiment by SC and HR, and the experiment was performed in a temperature-controlled room (22 °C). Coping behaviour was assessed only in the analogue trauma condition. Scenario-specific variables such as experience with computer games, sense of presence for VR and vividness for SDI as well as control questions for both paradigms (e.g. level of realness, compliance and attention) were measured after the experiment. Finally, participants completed the IMQ the following day and the IES-R one week later to assess intrusive memories, followed by a debriefing.

### 2.3 Materials

#### 2.3.1 Experimental and control stimuli

The analogue traumatic event in both paradigms comprised a multi-sensory simulation of an emergency scenario in an underground parking lot and in addition to visual presentation in VR, was presented auditorily via headphones and olfactorily by exuding the smell of smoke via a ventilator (Bordnick et al., 2005; Munyan et al., 2016). Participants were instructed to go to their virtual or imagined car. Shortly before reaching the car, a loud detonation followed by fading lights and the smell of atomised smoke were presented. A burning car and an injured man crying for help were then presented three-dimensionally in the VR scenario, or the participant was instructed to imagine this according to the SDI script. Psychophysiological threat was induced by a virtual presentation or the imagination of smoke and coughing sounds. The scenarios ended once the participants left the parking lot.

The non-stressful control situation consisted of a simulation (VR) or imagination (SDI) of the same underground parking lot but without any negative events happening. This was presented auditorily via headphones in both paradigms and three-dimensionally in the VR scenario. Participants were instructed to go to their virtual or imagined car that was momentarily blocked by an unloading pick-up truck. Meanwhile, they could search for and contact the driver.

##### 2.3.1.1 Virtual Reality (VR)

Based on a modified video-game simulator (Valve’s Source Engine) the neutral and analogue trauma scenarios in VR were presented visually in a first-person perspective using two colour displays of a Head Mounted Display (HMD; Type TriVisio VR Vision) in 3-D and olfactorily. Participants’ head movements were transmitted via a Calibri tracker in the HMD to record visual field changes within the VR. Participants were provided with a joystick (Type Thrustmaster T.16000M) to perform movements within the VR. We assessed previous experience with computer games. Participants received VR training before the virtual scenarios including the underground parking lot and the car for the experimental conditions. The time within the VR scenarios and coping behaviour were automatically registered by the game engine.

##### 2.3.1.2 Script-Driven Imagery (SDI)

The neutral and analogue trauma SDI situations were presented via audio script and olfactorily. The audio script comprised a high number of detailed adjectives to stimulate vivid mental images (Weidmann, Conradi, Groger, Fehm, & Fydrich, 2009) and was orally presented in the present tense by headphones with an appropriate emotional prosody to increase emotional engagement (Pell, Jaywant, Monetta, & Kotz, 2011). Participants received general mental imagery training (Blackwell et al., 2015) and a more specific mental imagery training including an introduction to the underground parking lot and the car used in the experimental conditions before the SDI scenarios. Participants were instructed to generate vivid mental images from a first-person perspective with their eyes closed and to experience the associated sensory responses and feelings as if they were actively involved while listening to the script (Holmes, Coughtrey et al., 2008). Participants were instructed to continue with the mental imagery and to report on their scenario-related feelings, thoughts and behaviour once the script terminated. The time spent within the SDI scenarios was recorded by the experimenter.

#### 2.3.2 Psychometric measures

##### 2.3.2.1 Psychopathology

Individual psychological distress related to overall psychopathology was assessed by the Global Severity Index (GSI) of the 53-item *Brief Symptom Inventory* (BSI; Franke, 2000) during the last week on a 5-point-scale (0 = *not at all* to 4 = *extreme*). The GSI has demonstrated high internal consistency, at *α* = 0.95 (Franke, 2000).

Current depressive symptoms were measured with the 20-item simplified *Beck Depression-Inventory* (BDI-S; Beck, Steer, & Brown, 1996; Hautzinger, Keller, & Kühner, 2006; Schmitt & Maes, 2000) on a 6-point scale (from 0 = *never* to 5 = *almost always).* The clinical cut-off value of the simplified version is 35 (Schmitt, Altstötter-Gleich, Hinz, Maes, & Brähler, 2006). The BDI-S has shown excellent reliability of *α* =0.93 (Schmitt et al., 2003).

Physiological and cognitive anxiety symptoms within the last 7 days were measured with the 21-item *Beck Anxiety Inventory* (BAI; Beck, Epstein, Brown, & Steer, 1988; Margraf & Ehlers, 2007) on a 4-point scale (0 = *not at all* to 4 = *extreme*). Internal consistencies in non-clinical samples have ranged between α = 0.76–0.94 (Margraf & Ehlers, 2007).

##### 2.3.2.2 Scenario-specific variables

The tendency to use visual mental imagery in daily life was measured with the 12-item *Spontaneous Use of Imagery Scale* (SUIS; Reisberg, Pearson, & Kosslyn, 2003) on a 5-point scale (1 = never to 5 = *always*). The SUIS has demonstrated high internal consistency, at α = 0.98 (Reisberg et al., 2003).

The sense of presence in the virtual environment was measured with the 14-item *IGroup Presence Questionnaire* (IPQ; Schubert, Friedmann, & Regenbrecht, 2001) with the subscales spatial presence (sense of being physically present), the experienced involvement and realism and the sense of being there on a 7-point scale (*0 = not at all to 6 = very much*). The internal consistency of the IPQ was α = 0.63–0.78 (Schubert, 2003).

Scenario-specific variables were further assessed with a questionnaire regarding previous experience with computer games for VR (1 = *not at all* to 6 = *extreme*), vividness (1 = *not at all* to 6 = *extreme)* and appropriate speed of the audio script for participants personal mental imagery (yes/no) for SDI as well as control questions, e.g. concerning the level of realness (1 = *not at all* to 6 = *extreme)*, compliance (1 = *not at all* to 6 = *extreme)* and attention (dichotomous scale (*yes/no)* for correct detail recognition) for VR and SDI.

##### 2.3.2.3 Subjective emotional responses

Anxiety, arousal and helplessness were rated on an 11-point Likert scale (0 = *not at all* to 10 = *very strong*) (Leung, 2011). Stress was measured with a visual analogue scale, ranging from 0-100.

##### 2.3.2.4 Intrusive memories

Spontaneous involuntary intrusive memories one day after the analogue trauma were measured with the modified 10-item *Intrusive Memory Questionnaire* (IMQ; Michael & Ehlers, 2007). The IMQ assessed the frequency (absolute number of occurrences) of and related worry (“0” = *not present* to “100” = *most extreme*) about intrusive memories across sensory modalities (visual images, sounds/smells) and thoughts, as well as mental occupation with the precipitating event (0–100%; relative amount of time of the total time since experiencing the event). In this study, we did not analyse the 3 items concerning temporal appearance of intrusive images, thoughts and sounds/smells. Internal consistency of the IMQ in the current study was α = 0.88.

The severity of intrusion, avoidance, and hyperarousal symptoms during the week after the analogue trauma were examined with the 22-item *Impact of Event Scale-Revised* (IES-R; (Maercker & Schützwohl, 1998; Weiss & Marmar, 1997) on a 4-point scale (0 = *not at all* to 4 = *often*). The IES-R has shown good reliability, at α = 0.79–0.90 (Maercker & Schützwohl, 1998).

#### 2.3.3 Coping behaviour assessment

For the VR method, coping behaviour was recorded automatically from the game engine. For the SDI method, participants were instructed to vividly imagine themselves being actively involved in the events and report their coping behaviour directly after SDI analogue trauma exposure. Based on expert ratings of professional firefighters from a local fire station, the following coping behaviours *activate fire alarm, approach emergency, take fire extinguisher*, *stoop over* (in order to protect oneself from smoke), *address person*, *extinguish fir*e and *take car exit or stairs* to leave the parking lot were classified as “adaptive behaviour”, while *take elevator* was classified as “maladaptive behaviour” for the given emergency situation. Furthermore, weighted average scores of each coping behaviour were obtained by experts’ rating regarding the importance (0 = *not important* to 10 = *very important*) of each behaviour occurring within the given emergency scenario (importance x frequency). In addition, a total score of adaptive and maladaptive behaviours was calculated for each individual. The total score was computed as the difference of adaptive and maladaptive behaviours (total sum score adaptive minus maladaptive values). Higher scores indicate more adaptive coping behaviour.

#### 2.3.4 Acquisition and analysis of the physiological data

We recorded skin conductance (SC) and heart rate (HR) as objective biological markers of autonomic activity and emotional arousal (Berntson et al., 1994; Boucsein, 2012). SC is strongly associated with emotional processes and functions as a sensitive indicator of emotional arousal regulated by the sympathetic nervous system (Boucsein, 2012; Critchley, 2002). As another established indicator of the autonomic stress response we used HR, reflecting the modulation of both the sympathetic and the parasympathetic nervous system (Berntson et al., 1994; Cacioppo, Uchino, & Berntson, 1994).

Both peripheral physiological parameters were recorded by Varioport II Systems (Becker Meditec, Karlsruhe, Germany). SCL was recorded at a sampling rate of 125 Hz, over two 11 mmAg/AgCl electrodes on the middle phalanx of the index and middle fingers of the immobilised non-dominant hand, a constant current flow voltage of 0.5 V was applied. To avoid movement artefacts, joystick operations were performed with the dominant hand. HR was recorded at a 400 Hz sampling rate using a 3-lead Wire electrocardiogram (ECG) via three dermal electrodes placed over the chest (*Einthoven's Triangle*). During the experiment, participants were monitored and instructed to avoid moving.

Subsequent data reduction and artefact editing of the physiological data parameters were performed using MATLAB-based Autonomic Nervous System Laboratory software (ANSLAB; Wilhelm & Peyk, 2005) in accordance with recommended procedures (Blechert, Peyk, Liedlgruber, & Wilhelm, 2016). To smooth the signals, a 1 Hz low-pass filter was applied to the SC data as well as a 40 Hz low-pass, 50 Hz notch and a 0.5 Hz high-pass filter to the ECG data. Average scores of SCL in microSiemens (μS) and heart rate in beats per minute (BPM) were exported at one-minute intervals to compute mean levels of skin conductance and heart rate over the middle 3 of the 5 min for each phase (baseline, neutral condition, analogue trauma condition).

### 2.4 Data analysis

Independent t-tests and chi-square tests were conducted to examine for any baseline differences in socio-demographic and scenario-specific variables. Paired sample t-tests were performed to test for differences in subjective (anxiety, arousal) and physiological (SC, HR) emotional response between baseline levels and analogue trauma conditions as a manipulation check.

A two-way mixed design MANOVA was applied with method (VR, SDI) as between-subjects factor and condition (neutral, analogue trauma) as within-subjects factor to investigate emotional reactivity (subjective: anxiety, arousal, stress, helplessness; physiological: SC, HR). To capture the emotional response to the experimental conditions and paradigms as well as to control for the individual baseline state levels before the conditions, we calculated a reactivity score by subtracting the mean levels (HR, SC) or subjective ratings of the baseline phase from the respective mean levels and subjective ratings of the neutral as well as the analogue trauma condition.

A one-way MANOVA was conducted to analyse the effect of the stress induction methods VR and SDI (between-group factor) on coping behaviour as well as on intrusive memories one day after analogue trauma (IMQ). Independent sample t-tests were performed to compare VR and SDI regarding intrusive memories, hyperarousal and avoidance (IES-R) one week after analogue trauma.

Effect sizes were reported (Cohen, 1988). A significance level of α = 0.05 was used for two-tailed conservative hypothesis testing.

## 3 Results

### 3.1 Sample characteristics

There were no differences in demographic characteristics, psychopathology symptoms or trait mental imagery between the VR and SDI groups (Table 1).

**Table 1:** Participant characteristics.

	Stress induction paradigm		Test statistic	*p*
	Virtual Reality (VR) (*n* = 66)	Script-Driven Imagery (SDI) (*n* = 61)		
Sex*, N* (%)			χ^2^(1, *N* = 127) = 0.56	0.813
female	52 (79)	47 (77)		
male	14 (21)	14 (23)		
Age (years): *M (SD)*	22.38 (4.14)	23.44 (7.37)	*t*(93) = 0.99	0.324
University qualification, *N* (%)	65 (98.5)	61 (100.0)	χ^2^ (1, *N* = 127) = 0.93	0.334
BSI - GSI: *M (SD)*	0.44 (0.40)	0.45 (0.44)	*t*(125) *=* 0.19	0.848
BDI-S: *M (SD)*	22.43 (11.98)	22.27 (13.68)	*t*(122) = 0.07	0.945
BAI: *M (SD)*	2.63 (3.60)	2.37 (3.70)	*t*(123) = 0.41	0.686
SUIS: *M (SD)*	38.35 (9.21)	38.23 (8.85)	t(124) = 0.07	0.941
Experience in rescue service, *N* (%)	3 (4.8)	3 (5.2)	χ^2^ (1, *N* = 120) = 0.01	0.933

*Note*. BSI-GSI = Brief Symptom Inventory-Global Severity Index; BDI-S = Beck Depression Inventory-S (simplified version; note that the clinical cut-off score of 35 is different from the original BDI/BDI-II); BAI = Beck Anxiety Inventory; SUIS = Spontaneous Use of Imagery Scale.

### 3.2 Scenario-specific variables

The analogue trauma exposure time for the total sample was 5 min (*M* = 5.26, *SD* = 2.54). We found that the speed of the audio script was mainly perceived as being appropriate for participants' personal mental imagery, high levels of vividness and perspective-taking from a first-person perspective in SDI as well as low levels of experience with computer games and symptoms of cyber sickness and high levels of presence in VR, thus reflecting a successful method implementation. There were no differences between VR and SDI in central characteristics such as motivation, immersion, trait mental imagery, perceived realness, avoidance and attention. Results for the scenario-specific variables are shown in the Supplementary Table A.1.

### 3.3 Manipulation check

Emotional responses to the baseline phase and analogue trauma condition were significantly different regarding subjective anxiety (*t*(124) = 13.44, *p* < 0.001, *d*_Z_ = 1.20) and arousal (*t*(123) = 12.14, *p* < 0.001, *d*_Z_ = 1.09) as well as physiological levels of emotional arousal indicated by SC (*t*(106) = 3.03, *p* = 0.003, *d*_Z_ = 0.91) and HR (*t*(106) = 9.40, *p* < 0.001, *d*_Z_ = 0.29) with higher levels in the analogue trauma condition for anxiety (*M*_BL_ = 0.31, *SD*_BL_ = 0.73 vs *M*_AT_ = 3.43, *SD*_AT_ = 2.63), arousal (*M*_BL_ = 1.84, *SD*_BL_ = 1.65 vs *M*_AT_ = 5.02, *SD*_AT_ = 2.63), SC (*M*_BL_ = 1.34, *SD*_BL_ = 0.57 vs *M*_AT_ = 1.76, *SD*_AT_ = 0.65) and HR (*M*_BL_ = 75.24, *SD*_BL_ = 11.46 vs *M*_AT_ = 78.78, *SD*_AT_ = 14.41), indicating a successful stress induction.

### 3.4 Psychophysiological emotional reactivity

Multivariate main effects for method (*F*(6,94) = 4.08, *p* = 0.001, η_p_^2^ =0.74) and condition (*F*(6,94) = 38.15, *p* < 0.001, η_p_^2^ =0.71) as well as method x condition interaction (*F*(6,94) = 14.43, *p* < 0.001, η_p_^2^ =0.48) were significant regarding psychophysiological emotional reactivity.

**Fig. 2:**
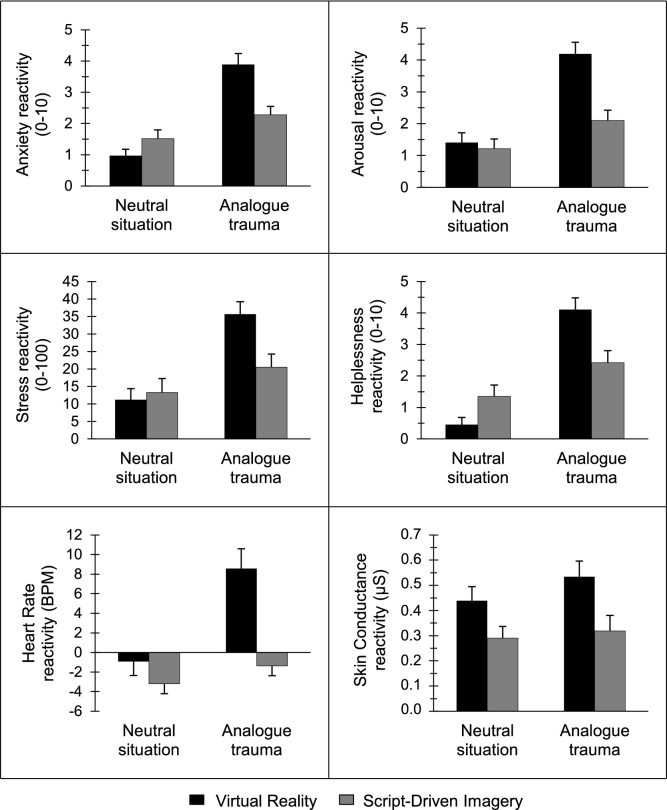
**Emotional reactivity in the neutral and analogue trauma condition as a function of experimental trauma induction paradigm**. Emotional responses of subjective anxiety, arousal, stress and helplessness as well as physiological arousal by heart rate in beats per minute (BPM) and skin conductance in microSiemens (μS) Virtual Reality or Script-Driven Imagery stress induction compared to the respective neutral situation condition. Significant effects were found for anxiety (Condition, Method x Condition), arousal (Condition, Method, Method x Condition), stress (Condition, Method x Condition), helplessness (Condition, Method x Condition), Heart Rate (Condition, Method, Method x Condition) and Skin Conductance (Condition, Method) reactivity. Error bars represent standard errors of the mean.

As displayed in Table 2 and Fig. 2, our results revealed significant main effects of method for arousal, SC and HR with a higher emotional reactivity in VR vs SDI in both the neutral and analogue trauma conditions and no significant differences between VR vs SDI for subjective anxiety, stress and helplessness. Significant main effects of stress condition indicated higher reactivity for subjective anxiety, arousal, stress, helplessness, SC and HR in the analogue trauma vs neutral condition in both paradigms.

**Table 2:** Emotional reactivity as a function of experimental trauma induction paradigm.

	Emotional Reactivity	*df*	*F*	*p*	η_p_^2^
Method			2.39	0.126	0.02
Condition	Anxiety	1/99	104.56	<0.001	0.51
Method x Condition			37.94	<0.001	0.28

Method			9.85	0.002	0.09
Condition	Arousal	1/99	88.00	<0.001	0.47
Method x Condition			25.04	<0.001	0.20

Method			2.24	0.138	0.02
Condition	Stress	1/99	76.98	<0.001	0.44
Method x Condition			27.10	<0.001	0.22

Method			0.81	0.369	0.01
Condition	Helplessness	1/99	96.16	<0.001	0.49
Method x Condition			32.92	<0.001	0.25

Method			4.82	0.030	0.05
Condition	Skin Conductance	1/99	6.18	0.015	0.06
Method x Condition			3.50	0.064	0.03

Method			11.63	0.001	0.11
Condition	Heart Rate	1/99	55.79	<0.001	0.36
Method x Condition			25.90	<0.001	0.21

*Note.* Subjective emotional reactivity was assessed by responses of anxiety, arousal stress and helplessness; physiological emotional reactivity was measured by skin conductance and heart rate responses.

Method x condition interaction effects were significant with a higher reactivity for subjective anxiety, arousal, stress, helplessness and HR in the neutral condition and SDI method as well as a higher reactivity in the analogue trauma condition and VR method. No method x condition interaction effect was found for SC.

### 3.5 Coping behaviour

During analogue trauma, out of the total sample 78.1% approached the emergency, 50.0% activated the fire alarm, and 47.6% took a fire extinguisher. Furthermore, 29.8% addressed the other person, 58.1% stooped over to protect oneself from smoke, and 19.4% extinguished the fire successfully. To leave the situation, 16.9% took an elevator while 41.1% took the stairs and 8.9% the car exit (for classification see 2.3.3).

**Fig. 3:**
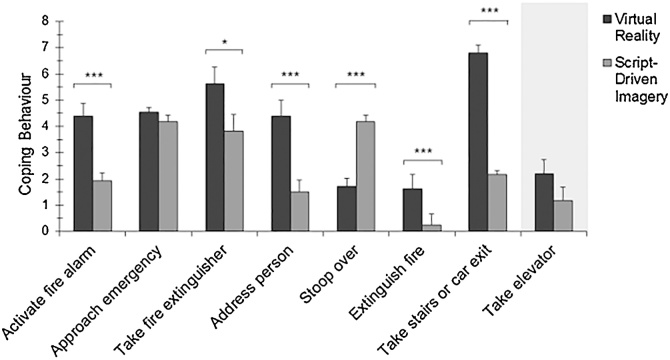
**Coping behaviour in the analogue trauma condition as a function of experimental trauma induction paradigm.** Adaptive coping behaviours and maladaptive coping behaviour (marked with a grey background) for the given emergency situation are displayed. Between-group differences in coping behaviour based on stress induction by Virtual Reality or Script-Driven Imagery are indicated (*p< 0.001**p < 0.01; ***p < 0.001). Error bars represent standard errors of the mean.

The multivariate main effect of method on coping behaviour was statistically significant (*F*(1,115) = 28.80, *p* < 0.001, η_p_^2^ =0.67). As shown in Table 3 and Fig. 3, differences were found between VR and SDI regarding *activate fire alarm*, *take fire extinguisher*, *address person*, *stoop over*, *extinguish fire* and *take stairs or car exit* with higher levels of coping behaviour in the VR paradigm, except for *stoop over*. Stress induction by VR or SDI was not significantly different regarding *approach emergency* and *take elevator*. In addition, the total score of adaptive and maladaptive coping behaviours was significantly different between VR and SDI (*t*(122) = 5.22, *p* < 0.001) with higher levels in VR (*M* = 31.25, *SD* = 14.72) than SDI (*M* = 19.17, *SD* = 10.58) indicating more adaptive coping behaviours in VR.

**Table 3:** Coping behaviour as a function of experimental trauma induction paradigm.

Coping Behaviour	*df*	*F*	*p*	η_p_^2^
Activate fire alarm	1/122	16.55	<0.001	0.12
Approach emergency	1/122	1.46	0.229	0.01
Take fire extinguisher	1/122	4.05	0.046	0.03
Address person	1/122	13.35	<0.001	0.10
Stoop over	1/122	39.77	<0.001	0.25
Extinguish fire	1/122	17.23	<0.001	0.12
Take stairs or car exit	1/122	37.91	<0.001	0.24
Take elevator	1/122	2.30	0.132	0.02

*Note*. All listed coping behaviours were classified as adaptive, except “Take elevator” which was classified as maladaptive in the given emergency situation.

### 3.6 Intrusive memories

One day after analogue trauma, on the IMQ all participants reported intrusive thoughts (*M* = 2.21, *SD* = 0.24), visual images (*M* = 1.82, *SD* = 0.17) and sounds/smells (*M =* 0.90, *SD* = 0.13) as well as worry about intrusive visual images (*M* = 8.98, *SD* = 1.21), thoughts (*M =* 8.06, *SD* = 0.98) and sounds/smells (*M* = 5.07, *SD* = 0.64). Participants reported being mentally occupied with the precipitating event for 8% of the time since they had experienced the event one day before (*M* = 8.27, *SD* = 0.71). Between paradigms, frequencies of intrusive images and levels of related worry were significantly higher in VR vs SDI, particularly for worry about intrusive images (Table 4).

**Table 4:** Analogue intrusive memories, hyperarousal and avoidance symptoms as a function of experimental trauma induction paradigm.

	Stress induction paradigm				
	Virtual Reality (VR)	Script-Driven Imagery (SDI)	Test statistic	*p*	ES η_p_^2^/d
	*M* (*SD*)	*M* (*SD*)			
IMQ					
Frequency of intrusive images	2.28 (2.36)	1.35 (1.34)	F(1/125) = 7.23	0.008	0.06
Frequency of intrusive thoughts	2.63 (2.97)	1.79 (2.23)	F(1/125) = 3.24	0.074	0.03
Frequency of intrusive sounds/smells	1.13 (1.85)	0.66 (0.94)	F(1/125) = 3.26	0.074	0.03
Worry about intrusive images (% of time)	13.12 (18.28)	4.83 (4.74)	F(1/125) = 11.79	0.001	0.09
Worry about intrusive thoughts (% of time)	7.26 (10.59)	8.85 (11.43)	*F*(1/125) = 0.66	0.418	0.01
Worry about intrusive sounds/smells (% of time)	4.31 (5.71)	5.83 (8.52)	*F*(1/125) = 1.41	0.238	0.01
Mental occupation with event/consequences (% of time)	9.21 (8.84)	7.33 (7.04)	*F*(1/125) = 1.74	0.189	0.01
IES-R					
Intrusion	3.22 (3.58)	2.61 (3.03)	t(101) = 0.93	0.356	0.18
Hyperarousal	1.61 (2.61)	2.37 (4.29)	t(78) = 1.07	0.289	0.22
Avoidance	3.91 (4.77)	5.92 (7.27)	t(82) = 1.64	0.104	0.33
Total score	8.74 (9.06)	10.90 (12.33)	t(101) = 1.02	0.311	0.20

*Note.* IMQ = Intrusive Memory Questionnaire; IES-R = Impact of event scale – Revised.

At the one-week follow-up assessment, on the IES-R all participants reported avoidance of stimuli related to the analogue trauma (*M* = 4.86, *SD* = 6.14), intrusions (*M* = 2.93, *SD* = 3.33) and hyperarousal (*M* = 1.97, *SD* = 3.51) with a mean total score of 9.77 (*SD* = 10.74). The levels of intrusion, hyperarousal and avoidance subscales as well as the total IES-R score were not significantly different between VR and SDI one week after analogue trauma (Table 4).

## 4 Discussion

We investigated and compared for the first time the impact of two standardised stress inductions using an experimental analogue of a trauma event in either VR or SDI (between subjects) versus a neutral condition (within subjects), on certain peri- and post-traumatic risk-factors including psychophysiological emotional response, coping behaviour and intrusive memories of the event. This real-time assessment of risk factors was performed during the multisensory (visual, ofactory, auditory) analogue of a traumatic event under controlled experimental conditions. Both the VR and the SDI analogue trauma paradigms induced a psychophysiological stress response, coping behaviour and intrusive memories, with a greater extent of these analogue symptoms in VR as predicted.

### 4.1 Psychophysiological stress response

In general, psychophysiological stress responses were higher for the analogue trauma compared to the neutral condition. Furthermore, subjective and physiological arousal (HR and SC) were also in general higher in VR than SDI for both the analogue trauma and neutral conditions, with a considerable increase in subjective arousal and HR in the analogue trauma condition. This is in line with research showing that both biological markers, SC and HR reflect autonomic activity and emotional arousal (Berntson et al., 1994; Boucsein, 2012). SC is primarily thought to be regulated by the sympathetic nervous system and thus an increase in SC indicates increased sympathetic activity (Boucsein, 2012; Critchley, 2002). An increase in HR may reflect an increase in sympathetic activity, a decrease in parasympathetic activity, or both because HR typically reflects the modulation of both the sympathetic and the parasympathetic nervous system (Berntson et al., 1994; Cacioppo et al., 1994). The finding that both, SC and HR, are increased in response to VR supports clearly increased sympathetic activity and, possibly decreased parasympathetic, activity.

In line with our previous work, anxiety, arousal, stress, helplessness and HR were generally lower in the neutral and generally higher in the analogue trauma condition, with considerably increased reactivity in VR compared to SDI. The relatively weaker emotional stress response in SDI might be due to the following reasons: a) more difficulties imagining unpleasant than pleasant events (Destun & Kuiper, 1999; Kealy, Kuiper, & Klein, 2006), b) less personally relevant and detailed mental imagery since no autobiographical references of a previous similar traumatic event of this type existed (Holmes & Mathews, 2005; Holmes, Mathews, Mackintosh, & Dalgleish, 2008) or c) by participants adopting a more verbal, instead of imagery-based, processing of the audio-verbally presented script (Holmes & Mathews, 2005; Ji et al., 2016). The low level of trait mental imagery in our sample could have led to a less detailed and vivid imagination of the situation in SDI, associated with decreased stress responses (Holmes & Mathews, 2005).

Stronger emotional reactivity in VR might be the result of the additional 3-D visual stimuli presentation (Dores et al., 2013) or more experienced interactivity with the virtual environment (Chittaro & Sioni, 2015). The applied olfactory stimuli seem to have contributed considerably to both analogue trauma simulations, probably via a higher level of experienced realness (Munyan et al., 2016; Riva et al., 2007).

### 4.2 Coping behaviour

In the total sample, coping behaviour included a high proportion of adaptive actions, which has been associated with more resilience and less PTSD symptom development (Thompson et al., 2018). This result corresponds to the behavioural pattern shown in real emergency situations (Canter, 1996). Thus, both paradigms seem to offer a possibility for testing and training emergency situations and related coping behaviour in an experimental setting.

Participants showed more adaptive coping behaviour in VR than SDI analogue trauma. This might be the consequence of experiencing the VR analogue trauma as highly arousing, which is associated with increased attention and preparedness to act (Freeman, Lessiter, Pugh, & Keogh, 2005). In addition, the presentation of visual cues in VR might have had increased adaptive coping behaviour by facilitating visual orientation since the visual sense is predominantly used in our daily life (e.g. Peponis, Zimring, & Choi, 1990; Posner, Nissen, & Klein, 1976). This is especially important given some previous research showing that behaviour in VR seems to be transferred into the real world (e.g. Yee, Bailenson, & Ducheneaut, 2009). The only adaptive coping behaviour displayed more frequently in SDI was stooping over in order to protect oneself from smoke. Surprisingly, this was more frequent if it was induced by olfactory stimuli only in SDI compared to combined visual and olfactory presentation in VR. The two paradigms did not differ in terms of dysfunctional coping behaviour. The fact that participants similarly approached the emergency in both paradigms could be related to information-seeking behaviour as an adaptive strategy to better manage the stressful situation (Canter, 1996; McConnell et al., 2010). This further indicates no difference in avoidance of the analogue traumatic event between VR and SDI, which is an important requirement for successful stress induction.

### 4.3 Intrusive memories

Both paradigms induced analogue intrusive memories one day after the analogue trauma. In line with previous studies, the frequency of intrusive visual images and thoughts was higher than the frequency of intrusive sounds/smells (Dibbets & Schulte-Ostermann, 2015; Krans et al., 2010; Schweizer et al., 2017). Increased intrusive memories in our study might be related to the relatively high levels of peri-traumatic arousal in our study. The mental occupation with the precipitating event in both groups was relatively long considering the nature of an analogue trauma. However, it may also reflect the timely proximity to the analogue trauma event, assuming more mental occupation with the event directly afterwards as a natural response and with a subsequent decline over time. Mental occupation following traumatic events can increase the risk of developing PTSD and maintain PTSD symptoms via maladaptive cognitions such as rumination. This may contribute to a sense of ongoing threat and avoidance of trauma-related stimuli, thus preventing trauma memory elaboration and integration (Dunmore, Clark, & Ehlers, 2001; Halligan, Michael, Clark, & Ehlers, 2003; Michael, Halligan, Clark, & Ehlers, 2007). Furthermore, high levels of intrusion-related worry were found in both groups, which has been associated with avoidance symptoms in PTSD (Wisco, Pineles, Shipherd, & Marx, 2013).

In particular, we found more frequent intrusive images and higher levels of related worry following VR compared to the SDI paradigm on the IMQ. This might be due to the mainly visual nature of intrusive memories in PTSD (e.g. Speckens, Ehlers, Hackmann, Ruths, & Clark, 2007) or because the VR analogue trauma was experienced as more stressful (Clark, Mackay, & Holmes, 2014; Ehlers & Clark, 2000), or more clearly multimodal. The considerably high level of worry about intrusive images might reflect an attempt to avoid the stressful images (Topper, Emmelkamp, Watkins, & Ehring, 2014).

After one week, the levels of intrusive memories decreased as measured by the IES-R with no differences between the paradigms.

### 4.4 Limitations

The analogue character of our study compared to a real trauma situation inherently limits the generalisation of our results to the real world, although the reaction patterns appear of a similar type to that in real traumatic situations (Kinateder et al., 2014; Rovira et al., 2009). In addition, the opportunities for behavioural coping actions were partly restricted in the analogue trauma situation, but this is also possible in a real emergency. Additionally, the type of emergency used for the analogue trauma (car park fire) may be infrequent in real life. More typical emergency situations, such as responding to a car accident, could be investigated in future studies.

### 4.5 Implications

Results suggest that certain peri- and post-traumatic risk factors can be readily investigated in real-time and from a first-person perspective under experimentally controlled conditions using only a 5-min simulation in VR and SDI to create an experimental analogue of a traumatic event. VR and SDI seem to be particularly useful laboratory paradigms to study risk factors in psychopathology development and adaptive coping as well as to identify and protect individuals at risk. The added olfactory stimuli demonstrated the potential of augmenting their efficacy.

Our results indicate that VR induces stronger analogue trauma stress reactions compared to the SDI. While VR is complex to perform and still relatively expensive, SDI is an economical alternative as an experimental stress induction. In the case of low levels of trait mental imagery, VR might be preferred to SDI due to the use of external images, which do not rely on participants’ memory or imagination ability (Eichenberg & Wolters, 2012; Weibel, Wissmath, & Mast, 2011). In contrast, a major strength of the SDI paradigm is its ability to flexibly adapt imagery content to an individual’s unique personal experience. This may be particularly useful if translation for example to a clinical setting using script driven imagery is used.

There are promising initial studies starting to use the VR approach to test potential treatment mechanisms (Cuperus, Laken, Van Den Hout, & Engelhard, 2016). As yet only a few real-time approaches exist that target peri- and post-traumatic stress responses in a clinical intervention against symptoms of post-traumatic stress (Cohen, Brinkman, Neerincx, & Ragan, 2016; Iyadurai et al., 2017; Nicholson et al., 2017; Pallavicini, Argenton, Toniazzi, Aceti, & Mantovani, 2016). Thus, having improved laboratory paradigms to aid intervention development via a focus on the underlying mechanims could be very useful (Holmes et al., 2018). That is, future studies aiming to prevent stress-associated psychopathology might benefit from the use of VR and SDI analogue trauma induction for developing preventive real-time interventions.

## 5 Conclusions

Our study showed that using a VR or SDI paradigm to create a multisensory analogue of a traumatic event in the laboratory allowed the investigation of peri- and post-traumatic factors that may confer risk for stress-associated disorders in real-time. Both paradigms induced psychophysiological stress responses, coping behaviour and intrusive memories, with a greater extent of these analogue symptoms with VR. VR and SDI may be further applied for the identification and protection of individuals who may be at clinical risk following exposure to a traumatic event. Both paradigms offer also novel experimental psychopathology models for studying trauma exposure and responses experimentally, and may provide important and valid insights about peri- and post-traumatic risk-factors. The development of future prevention and treatment applications might benefit from harnessing analogue trauma induction methods by VR or SDI, and in the future may allow us to perform peri- and post-traumatic real-time interventions under controlled settings.

## Funding

This research did not receive any specific grant from funding agencies in the public, commercial, or not-for-profit sectors.

## Conflicts of interest

The authors declare no conflicts of interest.

## References

[bib0005] Arble E., Lumley M.A., Pole N., Blessman J., Arnetz B.B. (2017). Refinement and preliminary testing of an imagery-based program to improve coping and performance and prevent trauma among urban police officers. Journal of Police and Criminal Psychology.

[bib0010] Bach P., Fenton-Adams W., Tipper S.P. (2014). Can’t touch this: The first-person perspective provides privileged access to predictions of sensory action outcomes. Journal of Experimental Psychology Human Perception and Performance.

[bib0015] Badour C.L., Feldner M.T. (2013). Trauma-related reactivity and regulation of emotion: Associations with posttraumatic stress symptoms. Journal of Behavior Therapy and Experimental Psychiatry.

[bib0020] Beck A.T., Epstein N., Brown G., Steer R.A. (1988). An inventory for measuring clinical anxiety: Psychometric properties. Journal of Consulting and Clinical Psychology.

[bib0025] Beck A.T., Steer R.A., Brown G.K. (1996). Manual for the Beck Depression Inventory-II.

[bib0030] Becker-Asano C., Sun D., Kleim B., Scheel C.N., Tuschen-Caffier B., Nebel B., S. D’Mello A., Graessner B., Schuller, Martin J.-C. (2011). Outline of an empirical study on the effects of emotions on strategic behavior in virtual emergencies. Affective computing and intelligent interaction.

[bib0035] Berntson G.G., Cacioppo J.T., Binkley P.F., Uchino B.N., Quigley K.S., Fieldstone A. (1994). Autonomic cardiac control. III. Psychological stress and cardiac response in autonomic space as revealed by pharmacological blockades. Psychophysiology.

[bib0040] Blackwell S.E., Browning M., Mathews A., Pictet A., Welch J., Davies J., Holmes E.A. (2015). Positive imagery-based cognitive Bias modification as a web-based treatment tool for depressed adults: A randomized controlled trial. Clinical Psychological Science: A Journal of the Association for Psychological Science.

[bib0045] Blechert J., Peyk P., Liedlgruber M., Wilhelm F.H. (2016). ANSLAB: Integrated multichannel peripheral biosignal processing in psychophysiological science. Behavior Research Methods.

[bib0050] Bonanno G.A., Westphal M., Mancini A.D. (2011). Resilience to loss and potential trauma. Annual Review of Clinical Psychology.

[bib0055] Bordnick P.S., Graap K.M., Copp H.L., Brooks J., Ferrer M. (2005). Virtual reality cue reactivity assessment in cigarette smokers. Cyber Psychology & Behavior.

[bib0060] Boucsein W. (2012). Electrodermal activity.

[bib0065] Brown P., Keenan J.M., Potts G.R. (1986). The self-reference effect with imagery encoding. Journal of Personality and Social Psychology.

[bib0070] Bryant R.A., Harvey A.G., Guthrie R.M., Moulds M.L. (2000). A prospective study of psychophysiological arousal, acute stress disorder, and posttraumatic stress disorder. Journal of Abnormal Psychology.

[bib0075] Bryant R.A., Harvey A.G., Guthrie R.M., Moulds M.L. (2003). Acute psychophysiological arousal and posttraumatic stress disorder: A two-year prospective study. Journal of Traumatic Stress.

[bib0080] Butler L.D., Blasey C.M., Garlan R.W., McCaslin S.E., Azarow J., Chen X.-H., Spiegel D. (2005). Posttraumatic growth following the terrorist attacks of September 11, 2001: Cognitive, coping, and trauma symptom predictors in an internet convenience sample. Traumatology.

[bib0085] Cacioppo J.T., Uchino B.N., Berntson G.G. (1994). Individual differences in the autonomic origins of heart rate reactivity: The psychometrics of respiratory sinus arrhythmia and preejection period. Psychophysiology.

[bib0090] Canter D.V. (1996). An overview of behaviour in fires. Psychology in action.

[bib0095] Chittaro L., Sioni R. (2015). Serious games for emergency preparedness: Evaluation of an interactive vs. a non-interactive simulation of a terror attack. Computers in Human Behavior.

[bib0100] Clark I.A., Mackay C.E., Holmes E. (2014). Low emotional response to traumatic footage is associated with an absence of analogue flashbacks: An individual participant data meta-analysis of 16 trauma film paradigm experiments. Cognition & Emotion.

[bib0105] Cohen J. (1988). Statistical power analysis for the behavioural sciences.

[bib0110] Cohen I., Brinkman W.-P., Neerincx M.A., Ragan E. (2016). Effects of different real-time feedback types on human performance in high-demanding work conditions. International Journal of Human-computer Studies.

[bib0115] Cortese B.M., Leslie K., Uhde T.W. (2015). Differential odor sensitivity in PTSD: Implications for treatment and future research. Journal of Affective Disorders.

[bib0120] Courtney C.G., Dawson M.E., Schell A.M., Iyer A., Parsons T.D. (2010). Better than the real thing: Eliciting fear with moving and static computer-generated stimuli. International Journal of Psychophysiology.

[bib0125] Critchley H.D. (2002). Review: Electrodermal responses: What happens in the brain. The Neuroscientist.

[bib0130] Cuperus A.A., Klaassen F., Hagenaars M.A., Engelhard I.M. (2017). A virtual reality paradigm as an analogue to real-life trauma: Its effectiveness compared with the trauma film paradigm. European Journal of Psychotraumatology.

[bib0135] Cuperus A.A., Laken M., Van Den Hout M.A., Engelhard I.M. (2016). Degrading emotional memories induced by a virtual reality paradigm. Journal of Behavior Therapy and Experimental Psychiatry.

[bib0140] Davis M.H., Hull J.G., Young R.D., Warren G.G. (1987). Emotional reactions to dramatic film stimuli: The influence of cognitive and emotional empathy. Journal of Personality and Social Psychology.

[bib0145] Destun L.M., Kuiper N.A. (1999). Phenomenal characteristics associated with real and imagined events: The effects of event valence and absorption. Applied Cognitive Psychology.

[bib0150] Dibbets P., Schulte-Ostermann M.A. (2015). Virtual reality, real emotions: A novel analogue for the assessment of risk factors of post-traumatic stress disorder. Frontiers in Psychology.

[bib0155] Dores A.R., Almeida I., Barbosa F., Castelo-Branco M., Monteiro L., Reis M., Castro Caldas A. (2013). Effects of emotional valence and three-dimensionality of visual stimuli on brain activation: An fMRI study. NeuroRehabilitation.

[bib0160] Dunmore E., Clark D.M., Ehlers A. (2001). A prospective investigation of the role of cognitive factors in persistent Posttraumatic Stress Disorder (PTSD) after physical or sexual assault. Behaviour Research and Therapy.

[bib0165] Ehlers A., Clark D.M. (2000). A cognitive model of posttraumatic stress disorder. Behaviour Research and Therapy.

[bib0170] Eichenberg C., Wolters C. (2012). Virtual realities in the treatment of mental disorders: A review of the current state of research. Virtual reality in psychological, medical and pedagogical applications.

[bib0175] Enochsson L., Isaksson B., Tour R., Kjellin A., Hedman L., Wredmark T., Tsai-Fellaender L. (2004). Visuospatial skills and computer game experience influence the performance of virtual endoscopy. Journal of Gastrointestinal Surgery.

[bib0180] Franke G.H. (2000). Brief Symptom Inventory (BSI).

[bib0185] Freeman J., Lessiter J., Pugh K., Keogh E. (2005). When presence and emotion are related, and when they are not. Proceedings of the Conference at Presence.

[bib0190] Gamberini L., Cottone P., Spagnolli a, Varotto D., Mantovani G. (2003). Responding to a fire emergency in a virtual environment: Different patterns of action for different situations. Ergonomics.

[bib0195] Halligan S.L., Clark D.M., Ehlers A. (2002). Cognitive processing, memory, and the development of PTSD symptoms: Two experimental analogue studies. Journal of Behavior Therapy and Experimental Psychiatry.

[bib0200] Halligan S.L., Michael T., Clark D.M., Ehlers A. (2003). Posttraumatic stress disorder following assault: The role of cognitive processing, trauma memory, and appraisals. Journal of Consulting and Clinical Psychology.

[bib0205] Hassabis D., Kumaran D., Vann S.D., Maguire E. (2007). Patients with hippocampal amnesia cannot imagine new experiences. Proceedings of the National Academy of Sciences.

[bib0210] Hautzinger M., Keller F., Kühner C. (2006). Das Beck Depressionsinventar II [The Beck Depression Inventory II].

[bib0215] Holmes E.A., Bourne C. (2008). Inducing and modulating intrusive emotional memories: A review of the trauma film paradigm. Acta Psychologica.

[bib0220] Holmes E.A., Mathews A. (2005). Mental imagery and emotion: A special relationship?. Emotion.

[bib0225] Holmes E.A., Mathews A. (2010). Mental imagery in emotion and emotional disorders. Clinical Psychology Review.

[bib0230] Holmes E.A., Ghaderi A., Harmer C.J., Ramchandani P.G., Cuijpers P., Morrison A.P., Craske M.G. (2018). The Lancet Psychiatry Commission on psychological treatments research in tomorrow’s science. The Lancet Psychiatry.

[bib0235] Holmes E.A., Coughtrey A.E., Connor A. (2008). Looking at or through rose-tinted glasses? Imagery perspective and positive mood. Emotion.

[bib0240] Holmes E.A., Mathews A., Mackintosh B., Dalgleish T. (2008). The causal effect of mental imagery on emotion assessed using picture-word cues. Emotion.

[bib0245] Hyman I.E., Pentland J. (1996). The role of mental imagery in the creation of false childhood memories. Journal of Memory and Language.

[bib0250] Iyadurai L., Blackwell S.E., Meiser-Stedman R., Watson P.C., Bonsall M.B., Geddes J.R., Holmes E.A. (2017). Preventing intrusive memories after trauma via a brief intervention involving Tetris computer game play in the emergency department: A proof-of-concept randomized controlled trial. Molecular Psychiatry.

[bib0255] James E.L., Lau-Zhu A., Clark I.A., Visser R.M., Hagenaars M.A., Holmes E.A. (2016). The trauma film paradigm as an experimental psychopathology model of psychological trauma: Intrusive memories and beyond. Clinical Psychology Review.

[bib0260] Ji J.L., Heyes S.B., MacLeod C., Holmes E.A. (2016). Emotional mental imagery as simulation of reality: Fear and beyond—A tribute to peter lang. Behavior Therapy.

[bib0265] Johnsen E.L., Lutgendorf S.K. (2001). Contributions of imagery ability to stress and relaxation. Annals of Behavioral Medicine.

[bib0270] Kaufman G.F., Libby L.K. (2012). Changing beliefs and behavior through experience-taking. Journal of Personality and Social Psychology.

[bib0275] Kealy K.L.K., Kuiper N.A., Klein D.N. (2006). Characteristics associated with real and made-up events: The effects of event valence, event elaboration, and individual differences. Canadian Journal of Behavioural Science/Revue Canadienne Des Sciences Du Comportement.

[bib0280] Kinateder M., Ronchi E., Nilsson D., Kobes M., Müller M., Pauli P., Mühlberger A., Krasuski A., Rein G. (2014). Virtual reality for fire evacuation research. Federated Conference on Computer Science and Information Systems..

[bib0285] Kleim B., Westphal M. (2011). Mental health in first responders: A review and recommendation for prevention and intervention strategies. Traumatology.

[bib0290] Krans J., Näring G., Holmes E.A., Becker E.S. (2010). “I see what you’re saying”: Intrusive images from listening to a traumatic verbal report. Journal of Anxiety Disorders.

[bib0295] Lang P.J. (1979). A bio-informational theory of emotional imagery. Psychophysiology.

[bib0300] Lang P.J., Bradley M.M., Cuthbert B.N. (2005). International affective picture system (IAPS): Affective ratings of pictures and instruction manual. Technical report A-6.

[bib0305] Lazarus R.S. (1993). Coping theory and research: Past, present, and future. Psychosomatic Medicine.

[bib0310] Leung S.-O. (2011). A Comparison of psychometric properties and normality in 4-, 5-, 6-, and 11-point likert scales. Journal of Social Service Research.

[bib0315] Maercker A., Schützwohl M. (1998). Erfassung von psychischen Belastungssfolgen: Die Impact of Event Skala - revidierte Version. Diagnostica.

[bib0320] Margraf J., Ehlers A. (2007). Beck Angst-Inventar (BAI). http://www.pearsonassessment.de/out/pictures/media/322401.pdf.

[bib0325] Mathews A., Ridgeway V., Holmes E.A. (2013). Feels like the real thing: Imagery is both more realistic and emotional than verbal thought. Cognition & Emotion.

[bib0330] McConnell N.C., Boyce K.E., Shields J., Galea E.R., Day R.C., Hulse L.M. (2010). The UK 9/11 evacuation study: Analysis of survivors’ recognition and response phase in WTC1. Fire Safety Journal.

[bib0335] McIsaac H.K., Eich E. (2004). Vantage point in traumatic memory. Psychological Science.

[bib0340] McMillan S.J., Lievrouw L.A., Livingstone S. (2002). Exploring models of interactivity from multiple research traditions: Users, documents, and systems. Handbook of New media.

[bib0345] McTeague L.M., Lang P.J., Laplante M.-C., Cuthbert B.N., Shumen J.R., Bradley M.M. (2010). Aversive imagery in posttraumatic stress disorder: Trauma recurrence, comorbidity, and physiological reactivity. Biological Psychiatry.

[bib0350] Michael T., Ehlers A. (2007). Enhanced perceptual priming for neutral stimuli occurring in a traumatic context: Two experimental investigations. Behaviour Research and Therapy.

[bib0355] Michael T., Ehlers A., Halligan S.L., Clark D.M. (2005). Unwanted memories of assault: What intrusion characteristics are associated with PTSD?. Behaviour Research and Therapy.

[bib0360] Michael T., Halligan S.L., Clark D.M., Ehlers A. (2007). Rumination in posttraumatic stress disorder. Depression and Anxiety.

[bib0365] Munyan B.G., Neer S.M., Beidel D.C., Jentsch F., Sayer N., Nugent S. (2016). Olfactory stimuli increase presence in virtual environments. PLoS One.

[bib0370] Nicholson A.A., Rabellino D., Densmore M., Frewen P.A., Paret C., Kluetsch R., Lanius R.A. (2017). The neurobiology of emotion regulation in posttraumatic stress disorder: Amygdala downregulation via real-time fMRI neurofeedback. Human Brain Mapping.

[bib0375] Olff M., Langeland W., Gersons B.P.R. (2005). Effects of appraisal and coping on the neuroendocrine response to extreme stress. Neuroscience and Biobehavioral Reviews.

[bib0380] Orr S.P., Lasko N.B., Macklin M.L., Pineles S.L., Chang Y., Pitman R.K. (2012). Predicting post-trauma stress symptoms from pre-trauma psychophysiologic reactivity, personality traits and measures of psychopathology. Biology of Mood & Anxiety Disorders.

[bib0385] Oulton J.M., Takarangi M.K.T., Strange D. (2016). Memory amplification for trauma: Investigating the role of analogue PTSD symptoms in the laboratory. Journal of Anxiety Disorders.

[bib0390] Ozer E.J., Best S.R., Lipsey T.L., Weiss D.S. (2003). Predictors of posttraumatic stress disorder and symptoms in adults: A meta-analysis. Psychological Bulletin.

[bib0395] Pallavicini F., Argenton L., Toniazzi N., Aceti L., Mantovani F. (2016). Virtual reality applications for stress management training in the military. Aerospace Medicine and Human Performance.

[bib0400] Pearson J., Naselaris T., Holmes E.A., Kosslyn S.M. (2015). Mental imagery: Functional mechanisms and clinical applications. Trends in Cognitive Sciences.

[bib0405] Pell M.D., Jaywant A., Monetta L., Kotz S.A. (2011). Emotional speech processing: Disentangling the effects of prosody and semantic cues. Cognition & Emotion.

[bib0410] Peponis J., Zimring C., Choi Y.K. (1990). Finding the building in Wayfinding. Environment and Behavior.

[bib0415] Pole N., Neylan T.C., Otte C., Henn-Hasse C., Metzler T.J., Marmar C.R. (2009). Prospective prediction of posttraumatic stress disorder symptoms using fear potentiated auditory startle responses. Biological Psychiatry.

[bib0420] Posner M.I., Nissen M.J., Klein R.M. (1976). Visual dominance: An information-processing account of its origins and significance. Psychological Review.

[bib0425] Reisberg D., Pearson D.G., Kosslyn S.M. (2003). Intuitions and introspections about imagery: The role of imagery experience in shaping an investigator’s theoretical views. Applied Cognitive Psychology.

[bib0430] Riva G., Mantovani F., Capideville C.S., Preziosa A., Morganti F., Villani D., Alcañiz M. (2007). Affective interactions using virtual reality: The link between presence and emotions. Cyberpsychology & Behavior: The Impact of the Internet, Multimedia and Virtual Reality on Behavior and Society.

[bib0435] Rovira A., Swapp D., Spanlang B., Slater M. (2009). The use of virtual reality in the study of people’s responses to violent incidents. Frontiers in Behavioral Neuroscience.

[bib0440] Saxon L., Makhashvili N., Chikovani I., Seguin M., McKee M., Patel V., Roberts B. (2017). Coping strategies and mental health outcomes of conflict-affected persons in the Republic of Georgia. Epidemiology and Psychiatric Sciences.

[bib0445] Schmitt M., Maes J. (2000). Vorschlag zur Vereinfachung des Beck-Depressions-Inventars (BDI). Diagnostica.

[bib0450] Schmitt M., Altstötter-Gleich C., Hinz A., Maes J., Brähler E. (2006). Normwerte für das Vereinfachte Beck-Depressions-Inventar(BDI-V) in der Allgemeinbevölkerung. Diagnostica.

[bib0455] Schmitt M., Beckmann M., Dusi D., Maes J., Schiller A., Schonauer K. (2003). Messgüte des vereinfachten Beck-Depressions-Inventars (BDI-V). Diagnostica.

[bib0460] Schöne B., Wessels M., Gruber T. (2017). Experiences in virtual reality: A window to autobiographical memory. Current Psychology.

[bib0465] Schubert T. (2003). The sense of presence in virtual environments: A three-component scale measuring spatial presence, involvement, and realness. Zeitschrift Für Medienpsychologie.

[bib0470] Schubert T., Friedmann F., Regenbrecht H. (2001). The experience of presence: Factor analytic insights. Presence.

[bib0475] Schweizer T., Schmitz J., Plempe L., Sun D., Becker-Asano C., Leonhart R., Tuschen-Caffier B. (2017). The impact of pre-existing anxiety on affective and cognitive processing of a Virtual Reality analogue trauma. PLoS One.

[bib0480] Solberg Ø., Birkeland M.S., Blix I., Hansen M.B., Heir T. (2016). Towards an exposure-dependent model of post-traumatic stress: Longitudinal course of post-traumatic stress symptomatology and functional impairment after the 2011 Oslo bombing. Psychological Medicine.

[bib0485] Speckens A.E.M., Ehlers A., Hackmann A., Clark D.M. (2006). Changes in intrusive memories associated with imaginal reliving in posttraumatic stress disorder. Journal of Anxiety Disorders.

[bib0490] Speckens A.E.M., Ehlers A., Hackmann A., Ruths F.A., Clark D.M. (2007). Intrusive memories and rumination in patients with post-traumatic stress disorder: A phenomenological comparison. Memory.

[bib0495] Tammy Lin J.-H. (2017). Fear in virtual reality (VR): Fear elements, coping reactions, immediate and next-day fright responses toward a survival horror zombie virtual reality game. Computers in Human Behavior.

[bib0500] Thompson N.J., Fiorillo D., Rothbaum B.O., Ressler K.J., Michopoulos V. (2018). Coping strategies as mediators in relation to resilience and posttraumatic stress disorder. Journal of Affective Disorders.

[bib0505] Topper M., Emmelkamp P.M.G., Watkins E., Ehring T. (2014). Development and assessment of brief versions of the Penn state worry questionnaire and the ruminative response scale. The British Journal of Clinical Psychology/The British Psychological Society.

[bib0510] Uhrig M.K., Trautmann N., Baumgärtner U., Treede R.-D., Henrich F., Hiller W., Marschall S. (2016). Emotion elicitation: A comparison of pictures and films. Frontiers in Psychology.

[bib0515] van der Heiden L., Scherpiet S., Konicar L., Birbaumer N., Veit R. (2013). Inter-individual differences in successful perspective taking during pain perception mediates emotional responsiveness in self and others: An fMRI study. NeuroImage.

[bib0520] Weibel D., Wissmath B., Mast F.W. (2011). Influence of mental imagery on spatial presence and enjoyment assessed in different types of media. Cyberpsychology, Behavior and Social Networking.

[bib0525] Weidmann A., Conradi A., Groger K., Fehm L., Fydrich T. (2009). Using stressful films to analyze risk factors for PTSD in analogue experimental studies –which film works best?. Anxiety, Stress, and Coping.

[bib0530] Weiss D.S., Marmar C.R., Wilson J.P., Keane T.M. (1997). The impact of event scale - revised (IES-R). Assessing psychological trauma and PTSD.

[bib0535] Wilhelm F.H., Peyk P. (2005). ANSLAB: Autonomic nervous system laboratory 4.0 [Computer software].

[bib0540] Wisco B.E., Pineles S.L., Shipherd J.C., Marx B.P. (2013). Attentional interference by threat and post-traumatic stress disorder: The role of thought control strategies. Cognition & Emotion.

[bib0545] Yee N., Bailenson J.N., Ducheneaut N. (2009). The Proteus effect. Communication Research.

