# Supplementary material for: Psychophysiological reactivity, coping behaviour and intrusive memories upon multisensory Virtual Reality and Script-Driven Imagery analogue trauma: A randomised controlled crossover study
Source: J Anxiety Disord. 2018 Oct;59:42–52. doi: 10.1016/j.janxdis.2018.08.005 (PMC6249992; doi:10.1016/j.janxdis.2018.08.005)
Supplement: Supplementary file 1 [file mmc1.pdf]

Appendix A. Supplementary material

Table A.1. Scenario-specific variables

	Stress induction paradigm		Test statistic	*p*
	Virtual Reality (VR) (*n* = 66)	Script-Driven Imagery (SDI) (*n* = 61)		
Exposure time (Min): *M (SD)*	5.47 (2.86)	5.04 (2.13)	$t(122) = 0.96$	.340
Stress intensity of the analogue trauma: *M (SD)* [a]	5.02 (1.03)	4.71 (1.14)	$t(118) = 1.56$	.122
Stress intensity of the neutral condition: *M (SD)* [a]	2.97 (1.39)	3.09 (1.33)	$t(118) = 0.48$	.635
Motivation to fully participate in the experiment (compliance): *M (SD)* [a]	5.39 (0.88)	5.34 (0.66)	$t(118) = 0.30$	.767
Degree of immersion into the situation: *M (SD)* [a]	4.61 (0.84)	4.78 (0.90)	$t(118) = 1.03$	.306
Degree of realness: *M (SD)* [a]	4.61 (0.78)	4.36 (1.12)	$t(101) = 1.42$	.159
Sense of control: *M (SD)* [a]	3.60 (1.36)	4.03 (1.18)	$t(117) = 1.88$	.062
Ability to act: *M (SD)* [a]	3.90 (1.17)	4.22 (1.22)	$t(118) = 1.47$	.143
Need to act: *M (SD)* [a]	5.42 (1.05)	5.59 (0.90)	$t(118) = 0.93$	.353
Perception of smell of smoke: *N* (%)	53 (85.5)	51 (87.9)	$\chi^2(1, N = 120) = 0.16$	.694
Immersion facilitated by smell of smoke: *N* (%)	48 (77.4)	43 (74.1)	$\chi^2(1, N = 120) = 0.18$	.675
Plausibility of the cover story: *N* (%)	51 (82.3)	51 (87.9)	$\chi^2(1, N = 120) = 0.76$	.384
Avoidance of the situation, *N* (%)	9 (14.5)	6 (10.3)	$\chi^2(1, N = 120) = 0.48$	.490
Attentional control task (correct detail recognition): *N* (%)	55 (88.7)	55 (93.2)	$\chi^2(1, N = 121) = 0.74$	.388

**Script-Driven Imagery**		
Speed of the audio-script perceived as appropriate, *N* (%)	-	53 (91.4)
Perspective, *N* (%)		
Analogue trauma		
Bystander/observer	-	6 (10.0)
First-person-perspective	-	54 (90.0)
Neutral condition		
Bystander/observer	-	7 (11.7)
First-person-perspective	-	53 (88.4)
Vividness, *M (SD)* [a]		
Analogue trauma	-	4.47 (1.08)
Neutral condition	-	4.37 (1.15)
**Virtual Reality**		
Experience playing computer games: *M (SD)* [a]	1.84 (1.18)	-
IPQ: *M (SD)* [b]		
Sense of being there	4.40 (1.53)	-
Spatial presence	4.24 (0.86)	-
Involvement	4.08 (0.79)	-
Experienced Realism	4.04 (0.78)	-
Degree of cyber sickness: *M (SD)* [a]	1.97 (1.32)	-

Note. IPQ = Igroup Presence Questionnaire; if it is not otherwise mentioned it refers to the experience related to the analogue trauma condition, [a] Likert-scale from 1 = *not at all* to 6 = *extreme*; [b] Likert-scale from 0 = *not at all* to 6 = *extreme*

$^{*}p < .05$. $^{**}p < .01$. $^{***}p < .001$.
